# Species‐specific behaviour and environmental drivers of trap interactions in wild ornamental fishes

**DOI:** 10.1111/jfb.70217

**Published:** 2025-09-07

**Authors:** Mar Pineda, Daiani Kochhann, Jose Lindoso Garrido Melo, Jan Lindström, Kathryn R. Elmer, Adalberto Luis Val, Shaun S. Killen

**Affiliations:** ^1^ School of Biodiversity, One Health & Veterinary Medicine, College of Medical, Veterinary and Life Sciences University of Glasgow Glasgow UK; ^2^ Centro de Ciências Agrárias e Biológicas Universidade Estadual Vale do Acaraú Sobral Brazil; ^3^ Nova Esperança, Puranga da Conquista Sustainable Development Reserve Manaus Brazil; ^4^ Laboratory of Ecophysiology and Molecular Evolution INPA: Brazilian National Institute for Research of the Amazon Manaus Brazil

**Keywords:** Amazon Basin, fisheries‐induced evolution, harvest‐associated selection, social behaviour, trapping

## Abstract

The harvest of animals from the wild is a pervasive selective force, especially in fisheries, where harvesting often targets individuals with specific traits. While most research has focused on large‐scale commercial or recreational fisheries, little attention has been paid to artisanal fisheries, particularly those targeting ornamental species. Furthermore, environmental factors such as temperature and oxygen levels influence the behaviour of fishes, such as boldness and sociability, but their role in the harvesting process remains poorly understood. Here, we used underwater video to examine how two ornamental Amazonian fishes, *Hemigrammus* sp. and *Copella nattereri*, interact with artisanal trap gear. We quantified the number of passes, inspections, entries and exits using latency to inspect and enter traps as proxies for boldness, and coefficients of dispersion (CDs) to assess sociability and group coordination. We found that the majority of fish that inspected traps did not enter them, and a given trap typically caught one species over the other. Overall, *Copella* were captured more frequently, but within individual trials there was no statistical difference in catch numbers between species. While both species inspected traps, *Hemigrammus* exhibited significantly more passes and a higher rate of inspection. Latency to inspect and enter traps did not differ between species but decreased with increasing temperature for both. *Hemigrammus* also displayed greater group coordination, with higher CD values across behaviours. Notably, temperature had opposing effects on coordination: for *Hemigrammus*, CD of inspections increased with temperature and CD of exits decreased, whereas for *Copella*, inspection CD decreased and exit CD increased. These findings reveal that different species interact with fishing gear in behaviourally distinct ways, influenced by environmental conditions. This highlights the potential for selective pressures to vary not only by species, but also with ecological context. Understanding such dynamics is critical for predicting how artisanal fisheries may shape behavioural traits in wild populations.

## 1 INTRODUCTION

The harvest of animals from the wild is a major selective force that can have important repercussions for evolution (Allendorf et al., 2008; Allendorf & Hard, 2009; Darimont et al., 2015, Darimont et al., 2009). From trophy hunting to harvesting for subsistence and population control, the capture of animals by humans has led to both direct and indirect selection in wild populations (Leclerc et al., 2017; Miller, 1957; Mysterud, 2011; Pigeon et al., 2016). Harvest‐associated selection has been especially well‐documented in commercial fisheries, particularly the impacts of increased mortality and non‐random removal of specific phenotypes, including those related to size and life‐history traits (Enberg et al., 2012; Heino et al., 2015). If the traits targeted by fisheries are heritable, this can lead to a phenomenon known as fisheries‐induced evolution (FIE) (Crespel et al., 2021; Heino et al., 2013; Heino et al., 2015). While the focus on FIE has largely been on size and maturation related traits (Enberg et al., 2012), there is now increasing evidence that behavioural traits can also be targets of selection, with individuals that are bolder or more sociable being removed from a population by recreational and commercial fisheries, and being selected against (Arlinghaus et al., 2017; Diaz Pauli et al., 2015; Hollins et al., 2019; Koeck et al., 2020). However, the mechanisms behind how or why specific behaviours are selected are currently not understood.

The methods used to capture fish can also be selective because different gear types tend to capture individuals with specific traits (Álvarez‐Quintero et al., 2021; Hollins et al., 2018). Active gear types such as trawls target groups of fish and are more likely to capture social species that have a higher tendency to shoal or social individuals that follow a leader into a trawl (Hollins et al., 2018). In contrast, passive gears such as traps rely on individuals encountering and voluntarily interacting with a gear. The behaviour of fish around traps has been well‐documented in commercial food fisheries (Thomsen et al., 2010; Winger et al., 2016). Baited traps capture individuals with greater food‐searching behaviour (Thomsen et al., 2010), which is often linked to a higher metabolic demand (Killen et al., 2011). Similarly, passive gears have been shown to attract bolder individuals (Arlinghaus et al., 2017; Biro & Dingemanse, 2009; Diaz Pauli et al., 2015; Wilson et al., 1993), although how this relates to capture is less understood. For example, bolder fish may encourage other social individuals to enter a trap (Hollins et al., 2018), whilst more aggressive or territorial individuals may guard the entrance of a trap or deter others (Finucci et al., 2019; Winger et al., 2016), indicating the importance of recognising differences in behaviour among and between species. Abiotic factors such as light (Hedgärde et al., 2016), current (Folkins et al., 2021), temperature (Hollins et al., 2021; Thomsen et al., 2010) and hypoxia (Thambithurai et al., 2019) further affect behaviour during the capture process. Elevated temperature increases activity, which can lead to more encounters with gear. Increasing temperature also influences metabolic rate (Biro & Stamps, 2010), leading to a greater food demand (Killen et al., 2011) as well as an increase in boldness that can increase the likelihood of finding and entering a gear (Winger et al., 2016). Conversely, hypoxia can reduce appetite and lead to a reduction in foraging behaviour (Chabot & Claireaux, 2008; Killen et al., 2012), which could lead to a decrease in gear encounter rates. Furthermore, while the behaviour of fish around commercial traps and trawls is well observed, this knowledge is absent for many smaller‐scale artisanal fisheries.

The ornamental fishing industry is a multi‐billion dollar industry that involves the collection and distribution of fishes for the aquarium industry across the globe (Ojelade et al., 2024; Saxby et al., 2010). The trade is estimated to handle up to 1.5 billion individual fish per year (Evers et al., 2019; Stevens et al., 2017), including 4500 freshwater species (Miller‐Morgan, 2009), although estimates vary widely for the number of individuals collected and transported, and the consequent profits made (Evers et al., 2019; King, 2019; Stevens et al., 2017). It is also estimated that 10% of freshwater fishes used in the ornamental trade are harvested from the wild (Evers et al., 2019; OATA, 2016). The state of Amazonas, in Brazil, is often used as a case study for the capture of wild‐caught ornamental fishes (Evers et al., 2019; OATA, 2016;Tribuzy‐Neto et al., 2021; Zehev et al., 2015), with the trade contributing up to 80% of the local economy and supporting livelihoods (Tribuzy‐Neto et al., 2021; Zehev et al., 2015). A large variety of Amazonian species are caught using artisanal gears, including *cacuri*, a passive, non‐destructive trap designed to catch fish alive (Phang et al., 2019). While cacuri offer less precision when targeting commercially important species, they are typically favoured for their low environmental impact, affordability and suitability for capturing small bodied species, particularly in areas that are harder to access (Ferreira & Yamamoto, 2017; Ladislau et al., 2020; Phang et al., 2019). However, little is known about how specific species behave around cacuris or how environmental factors such as water temperature and dissolved oxygen, which both fluctuate widely in Amazonian ecosystems (Marengo et al., 2018; Val & De Almeida‐Val, 1995), influence capture dynamics. These environmental variables are known to affect behavioural traits such as sociability and boldness (Pineda et al., 2020; Tiddy et al., 2024), which may in turn mediate vulnerability to capture.

Despite the ecological and economic significance of the ornamental trade, the behaviour of ornamental species in the wild has rarely been studied, particularly in the context of selective harvest. In this study, we focus on two abundant Amazonian species, *Copella nattereri* and *Hemigrammus* sp., which are commonly found schooling in shallow floodplain habitats and frequently caught by artisanal fishers (Tribuzy‐Neto et al., 2021). Although they are not the primary targets of ornamental fishers, they are still among the most commonly exported (Tribuzy‐Neto et al., 2021). Additionally, their schooling behaviour, abundance and accessibility make them suitable for behavioural observation and analysis of species‐specific responses to fishing gear. While both species have a similar temperature tolerance (Dos Beltrão Anjos et al., 2017; Fróis et al., 2021), they both inhabit similarly oxygen‐ and temperature‐variable environments. This variation can also have important implications for selection as trait variation and heritability changes across environments, potentially amplifying or weakening harvest‐associated selection (Thambithurai et al., 2019). Additionally, given the rapid generation times of these small Amazonian species, they represent a valuable model for investigating how selection can operate in wild populations (Gordon et al., 2015).

By capturing underwater footage of Amazonian fishes during the trapping process, we provide a low‐cost method for observing behaviours of individuals in the wild. We also examine differences between species in their interactions with a passive gear type, including decisions to inspect, as well as decisions to bypass, a behaviour usually not examined by fisheries simulations (Hollins et al., 2019; Thambithurai et al., 2018, 2022). By linking this with environmental data, we examined the impacts of abiotic factors that affect behaviour, such as temperature and dissolved oxygen levels (Pineda et al., 2020; Tiddy et al., 2024). Specifically, we aimed to address the following questions: (1) what behavioural patterns and processes underlie the likelihood of fish being captured when in proximity to a trap; (2) is there a difference between species in the behaviours observed; and (3) do environmental factors influence the behaviours of individuals around a trap?

## 2 METHODS

### 2.1 Sampling area

**FIGURE 1:**
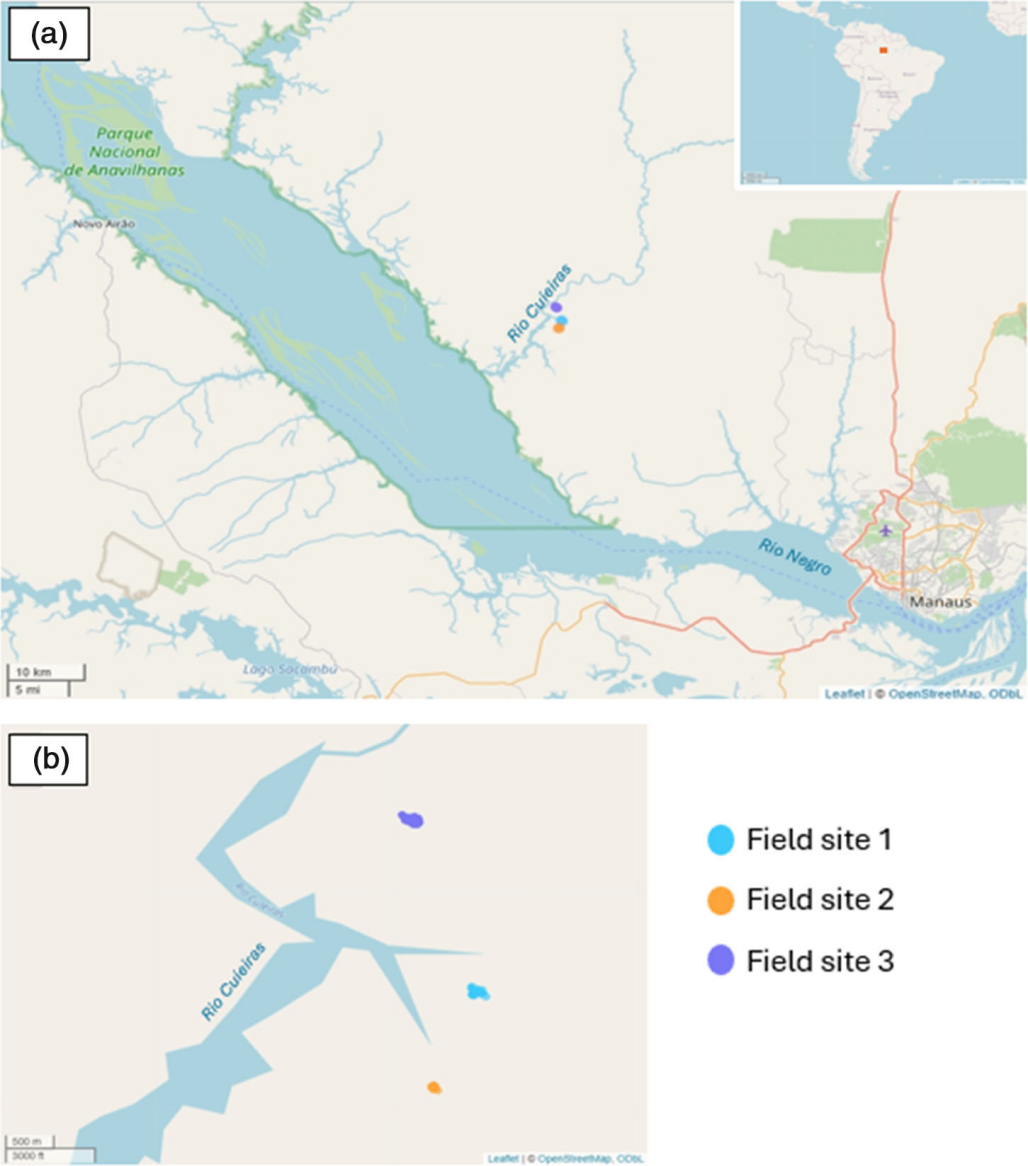
Map of field sites used for fishing: (a) area showing landmarks such as Manaus and the Rio Negro as well as an inset map of its location within Brazil; (b) area zoomed in to show where surveying took place within the three different field sites along igarapes of the Rio Cuieiras. The shape of the tracks corresponds to live data of our movements within the site. Maps were made using downloaded gpx data visualised with the leaflet package in R (Cheng et al., 2024).

Fieldwork was conducted within the Puranga da Conquista Sustainable Development Reserve (SDR) in the Brazilian Amazon. The SDR was created in 2014 in the Rio Negro Basin and is made up of approximately 77,000 hectares with 15 communities living within the protected area (Instituto Socioambiental, n.d.). Here, fishing is only permitted for subsistence for members of the community or for researchers with a permit and there is no history of fishing for the aquarium trade. Fishing occurred during the dry season, which is when ornamental fishers catch fish for the ornamental trade (da Silva Ladislau et al., 2021). In the dry season, floodplains dry into smaller streams known as *igarapés* and the capture of fishes becomes easier in these smaller streams. Fishing took place at three field sites from 15 to 19 September 2022 (Figure 1 and Table S1).

### 2.2 Sampling method

**FIGURE 2:**
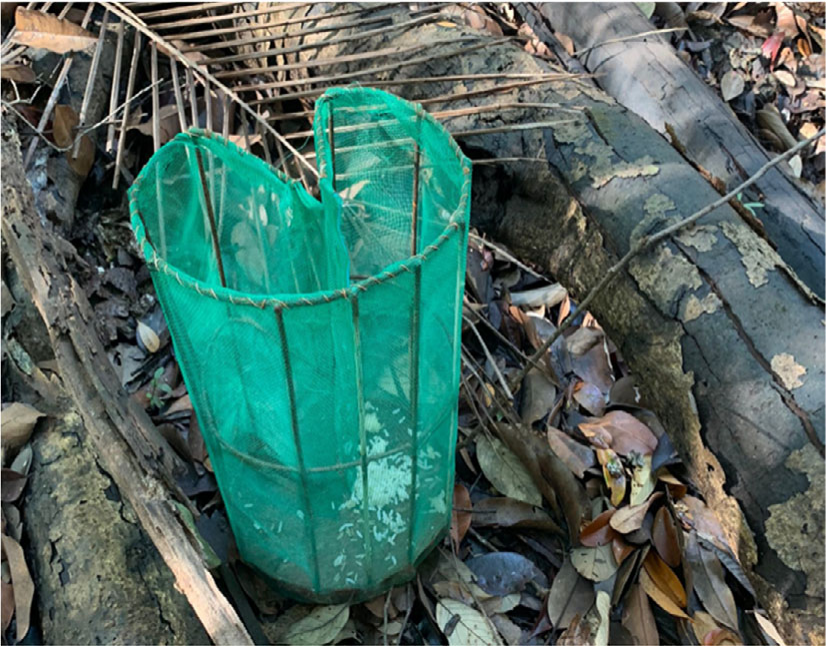
Cacuri used for trapping fish consisting of a metal frame (43 cm H, 22 cm diameter) covered in netting (<1.5 mm mesh size) attached to a wooden base with an inverted funnel entrance (6 cm diameter). Photograph by M. Pineda.

Fish were caught using an artisanal trap known as a cacuri (Figure 2). Traps were placed so that the top of the trap emerged from the surface of the water, with water depth varying between sites, and the traps were baited with rice or manioc powder. The process for capture was guided by our partner fisher and the trapping process closely mimicked that of the ornamental fishing trade.

Each day, traps were placed within a field site 6 to 14 times, resulting in 42 trapping trials across the five days and three sites. Traps were placed in pairs, with the distance between traps varying according to environmental constraints. To reduce the likelihood of behavioural interference, traps were typically positioned 10 m apart and trap locations were not reused across trials. The studied species were trapped outside of the breeding season so were not considered to be territorial, reducing the risk of interference. Cameras (GoPro Hero 4, Paralenz) were opportunistically placed at the entrance of traps to record the capture process, collecting footage for 36/42 trials. The cameras were operated manually by starting videos when they were placed in position. The videos then lasted 30 min. Environmental data (Figures S1 and S2: water temperature, air temperature, pH, oxygen saturation (% and mg/L) were also collected at each trap location using a YSI meter (YSI Pro20) as well as time of day (date and time).

The experimental procedures were approved by the Animal Use Ethics Committee of the Brazilian National Institute for Research of the Amazon (CEUA‐INPA), number 01280.000209/2018‐74. The permit for the collection of the biological material to carry out the research was authorised by the Brazilian Institute of Environment and Renewable Natural Resources (IBAMA/SISBIO), number 29837‐13.

### 2.3 Video analysis

Video analysis was done manually using Solomon Coder (version 19.08.02), which allows for the coding of behavioural events across the duration of a trial. The videos featured two of the most abundant species in the area, *Copella nattereri* and *Hemigrammus* sp., hereby referred to as Copella and Hemigrammus, respectively, which are both social schooling species. Two distinct forms of Hemigrammus were observed in the videos, although species‐level identification was not possible. These forms were visually distinguishable and are referred to here as Hemigrammus sp. 1 and Hemigrammus sp. 2. Hemigrammus sp. 1, the most abundant form, had a red coloration in the upper region of the eye and a clearly marked lateral line. Hemigrammus sp. 2 exhibited a smaller red area around the eye and a large black spot on the caudal peduncle. Behavioural analysis was conducted using Hemigrammus sp. 1 only, while sp. 2 is included in the taxonomic list (Table S2).

**FIGURE 3:**
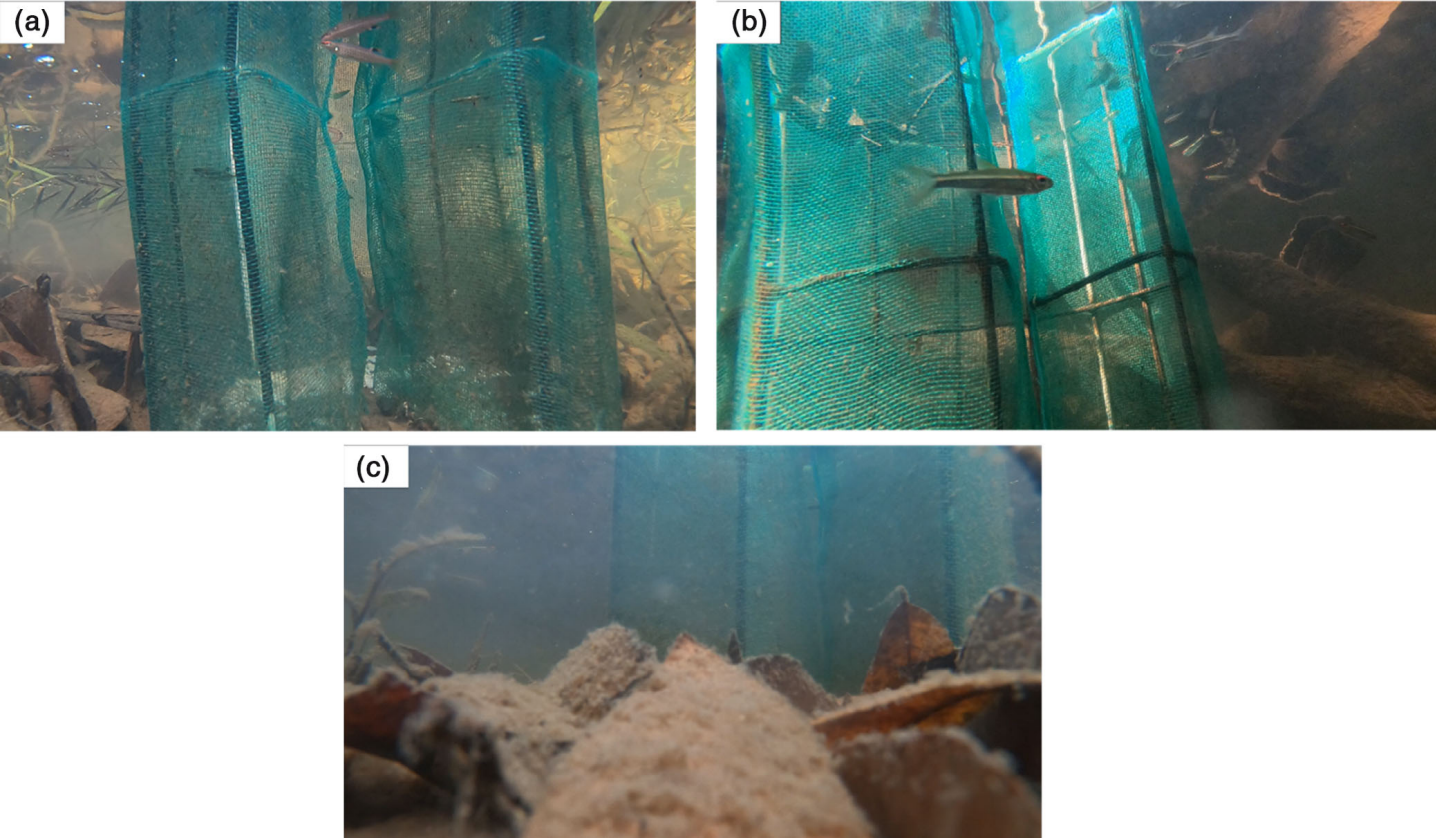
Visibility classifications for trap videos: (a) high visibility, the entire trap can be seen from top to bottom; (b) medium visibility, the top of the trap can be seen but not the bottom; (c) low visibility, both the top and the bottom of trap cannot be seen, and view is partially obscured by leaves and sediment.

Videos were categorised into visibility type (high, medium or low) to establish a criterion for determining suitability for behavioural analysis. The classification was based on the quality of the recording and how much of the trap was visible in each recording: high visibility videos showed the entirety of the trap, medium visibility videos showed a portion of the trap and low visibility videos were largely obstructed or poorly lit (Figure 3a–c). Low visibility videos typically lacked sufficient light or clarity to reliably identify species or code fish behaviours and were therefore excluded from behavioural analysis to preserve data quality. This resulted in nine high, 13 medium, 14 low videos.

The number of passes, inspections, entries and exits was recorded for each species at each trap as well as the time each behaviour happened in the video. Descriptions for each behaviour are shown in Table 1.

**TABLE 1:** Description of each behaviour and how it was classified.

Behavioural measure	Description
Pass	Whenever an individual passes by any visible part of the trap without inspecting it.
Inspect	Whenever an individual approaches the trap, touches the trap or interacts with any fish on the inside of the trap either at the entrance or any other visible part of the trap.
Enter	Whenever an individual enters the trap completely.
Exit	Whenever an individual exits the trap completely.
Latency to inspect	Time taken until the first fish of each species inspects the trap (seconds).
Latency to enter	Time taken until the first fish of each species enters the trap (seconds).
Rate of inspection	Rate of inspection within each trial for each species calculated within a specific time interval. Later averaged per trial for modelling.
Rate of entry	Rate of entry within each trial for each species calculated within a specific time interval. Later averaged per trial for modelling.
Sociability	Whether or not behaviours are clustered in time.

The location in which fish entered the trap was also recorded and was classified as either top, middle or bottom relative to the surface of the water column. Analysis was completed for all high visibility videos first. Preliminary analysis found that most individuals entered near the top of the trap (Figure S3). Subsequently, medium visibility videos were then analysed if the top of the trap was visible. Videos that were classified as low visibility were not used for video analysis or subsequent statistical analysis. Any videos that were less than 30 min long were also discarded. This resulted in 18 30‐min videos used overall for video and statistical analysis.

Further behaviours were then analysed manually. The latency of the first individual of each species to inspect and enter the trap was used as a proxy for boldness, with bolder individuals inspecting or entering the trap sooner. The number of individuals of each species retained by a trap at the end of the trial was calculated by subtracting the number of exits from the number of entries. The rate of inspection and rate of entry of each species was also calculated within each trial by dividing the time in the 30‐min videos into 60‐s time intervals. The number of behavioural events (either inspections or entries) were recorded within the interval to provide a behavioural count. The rate of the behaviour (inspection or entry) was then calculated as rate of behaviour = behavioural count/60 to convert the raw count of inspections into a rate of behaviour per second.

The sociability of each species was then analysed by calculating the coefficient of dispersion (CD) for each behaviour in the 30‐min trial (Chapman & Chapman, 1994; Hollins et al., 2019; Killen et al., 2018; Pineda et al., 2020). Each trial was split into 10‐s intervals and the number of behaviours within each time bin were counted. CD was then calculated as the variance/mean ratio across the intervals, with CD ≈ 1 indicating events were randomly distributed, CD < 1 indicating events were evenly spread, and CD > 1 indicating a clustering of events.

### 2.4 Statistical analyses

All statistical analyses were carried out using R version 4.2.2 (R Core Team, 2022). Linear models were used to test the effects of species, site, date and environment on the behaviours observed in the 42 trapping trials. The response variables were the frequency of passes, inspections, entries and exits, the number of individuals captured, inspection latency, entry latency, sociability, average rate of inspection, average rate of entry and the CD of each behaviour. For all models, the explanatory variables were species, site, date, water temperature and oxygen saturation as well as all relevant two‐way interactions. Initial models included any main predictors that were not correlated and model selection occurred via a stepwise approach based on Akaike Information Criterion (AIC) values using the MASS package (Ripley & Venables, 2009). The final model was selected when no further improvement in AIC could be made. In the results section, *p* values are described as suggested by Muff et al. (2022), which classifies significance thresholds as ‘little to no evidence’ (*p* > 0.1), ‘weak evidence’ (0.05 < *p* < 0.1), ‘moderate evidence’ (0.01 < *p* < 0.05), ‘strong evidence’ (0.001 < *p* < 0.01) and ‘very strong evidence’ (*p* < 0.001).

## 3 RESULTS

### 3.1 Number of fish captured

**FIGURE 4:**
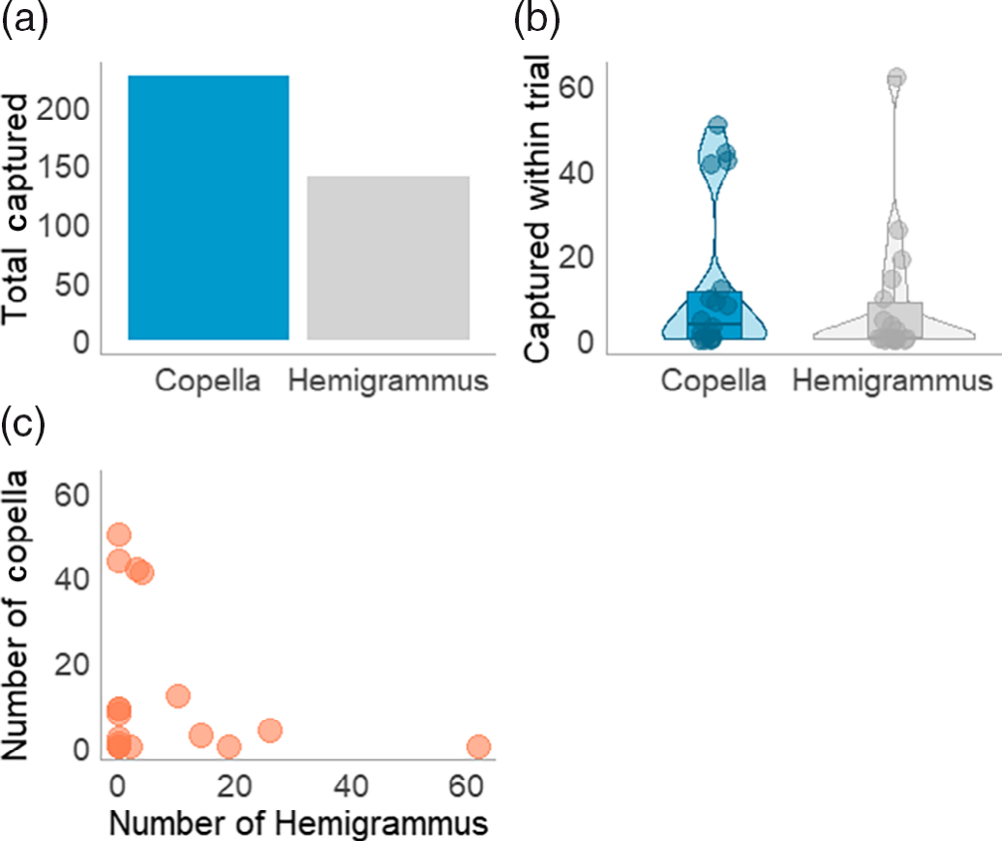
Number of fish captured by traps: (a) total number of individuals captured of each species across the field season; (b) total number of individuals captured of each species within each trial (here, each data point represents a trial); (c) the relationship between the number of individuals of each species caught within a trial.

Overall, while more captures were observed for Copella compared to Hemigrammus across trials (Figure 4a), there was no statistical evidence to support a difference in the number of individuals captured between species within a trial (Figure 4b). There was weak evidence to suggest that date of capture influences the number of individuals caught within a trial (*t* = −1.725, *p* = 0.095; Table S3). However, comparing the number of individuals captured from each species within a trial revealed that, within a given trial, one species would often be caught in a greater amount than the other, that is more Copella and fewer Hemigrammus, or vice versa (Figure 4c).

### 3.2 Frequency of behaviours

**FIGURE 5:**
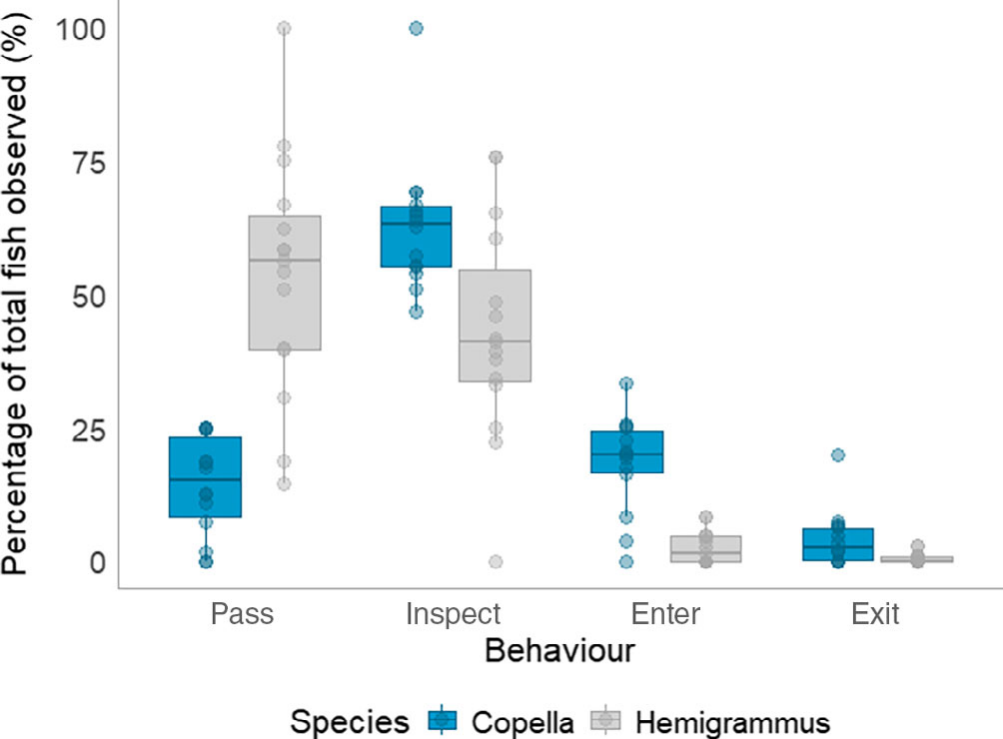
The number of passes, inspections, entries and exits observed as a percentage of total behaviours. Each data point represents an individual trial.

Over 5000 behavioural events were coded during video analysis. Overall, the number of passes and inspections were greater than the number of entries and exits for both species (Figure 5). Furthermore, there was a difference in the total number of inspections leading to entries between species (*t* = 5.252, *p* < 0.001), with 34.5% of inspections resulting in entries for Copella and only 9.3% of inspections resulting in entries for Hemigrammus. Furthermore, 27.6% of Copella that entered a trap exited, as did 22.7% of Hemigrammus.

While there was strong evidence that both date of capture (*t* = −2.151, *p* = 0.04) and species (*t* = 3.821, *p* < 0.001) explained variation in the number of observed passes, there was weak evidence for date and species explaining variation in the number of inspections, entries and exits (Figure S4 and Table S3).

### 3.3 Inspection and entry latency

**FIGURE 6:**
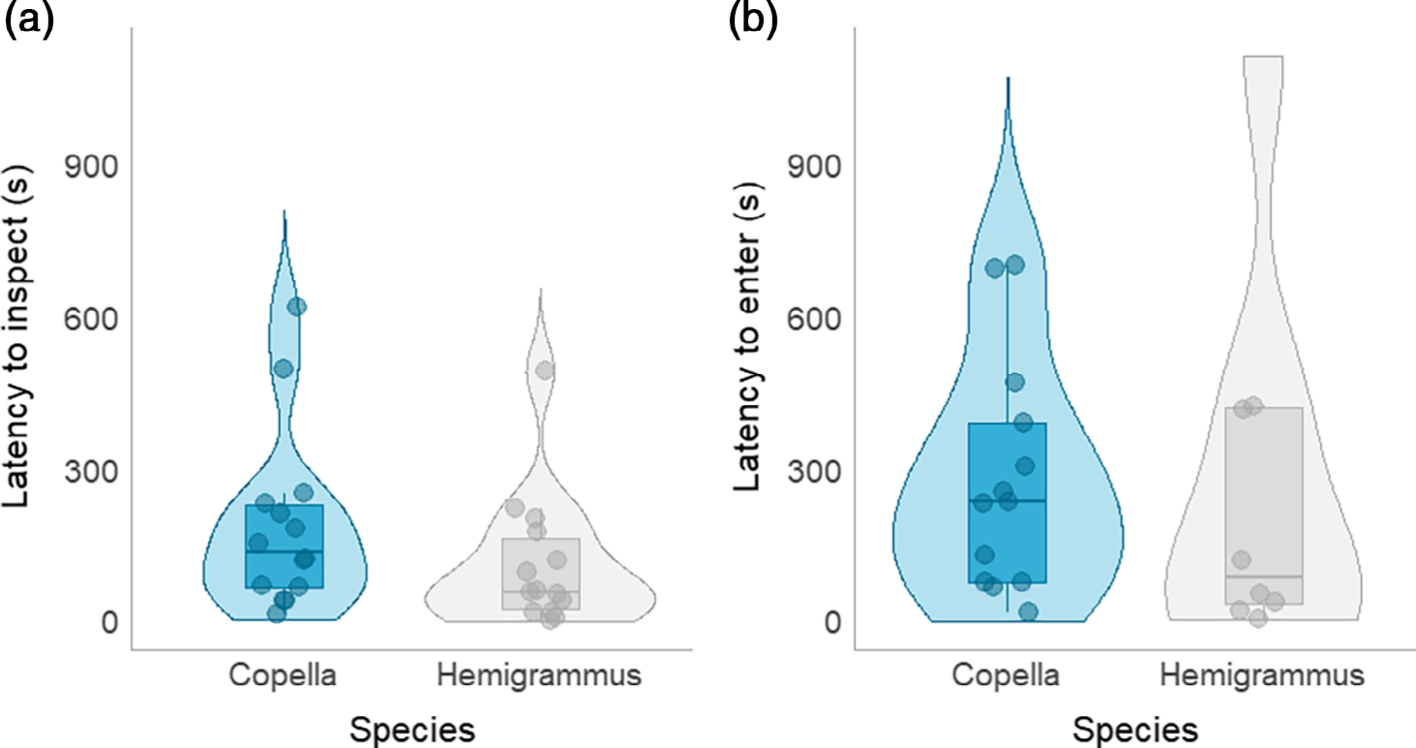
Inspection and entry boldness of Copella and Hemigrammus. Each individual data point corresponds to the first individual to inspect or enter within a trial: (a) time taken for the first individual within a trial to inspect a trap; (b) time taken for the first individual within a trial to enter a trap.

While the median inspection latency was lower for Hemigrammus compared to Copella, there was no evidence to suggest an overall difference between species in the latency of inspections (two‐sample *t*‐test: *t* = 1.289, *p* = 0.210) nor entries (two‐sample *t*‐test = *t* = 0.048, *p* = 0.963) due to the large amount of within‐species variation (Figure 6 and Table S4).

**FIGURE 7:**
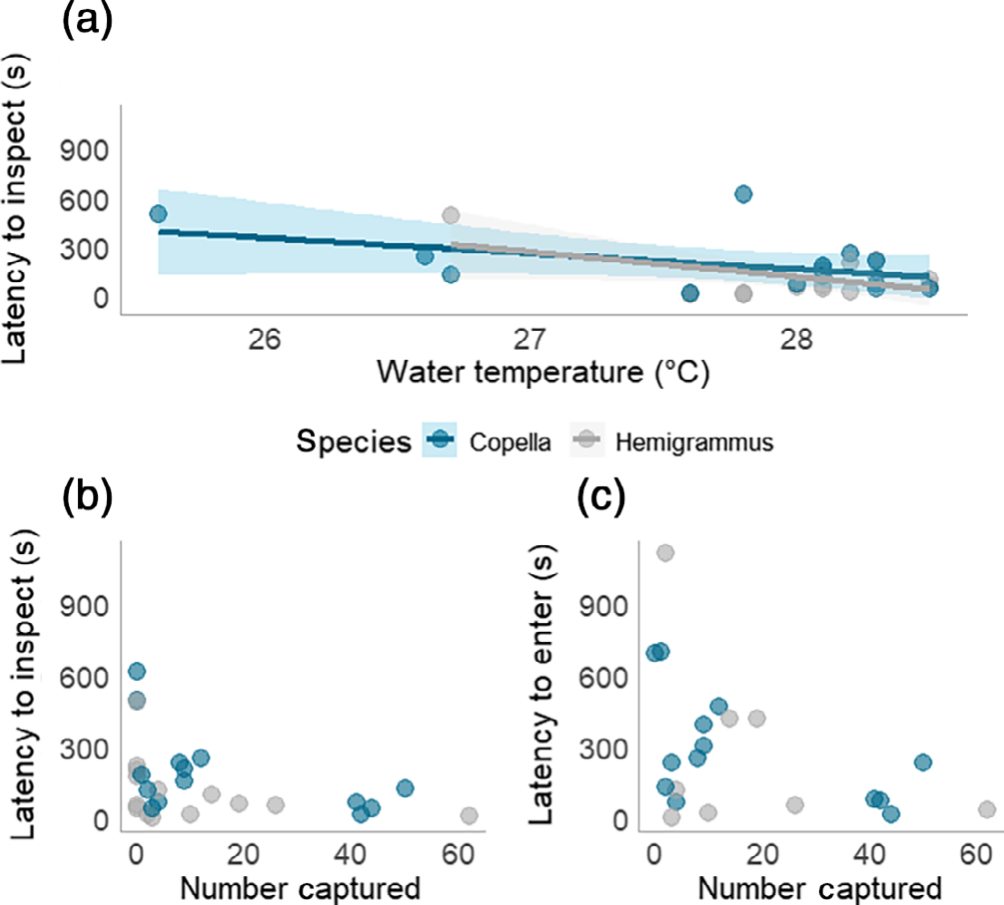
Factors affecting latency to inspect and enter a trap: (a) the relationship between latency to inspect and water temperature; (b) the time taken for the first individual to inspect a trap against the number of individuals captured; (c) the time taken for the first individual to enter a trap against the number of individuals captured. Each data point represents an individual trial and each data point is coloured according to species.

There was strong evidence that inspection latency decreased at higher temperatures (*t* = −2.969, *p* = 0.006; Figure 7a). Furthermore, while there did appear to be an interaction between water temperature and species, there was no statistical evidence for this (*t* = −0.816, *p* = 0.425). Additionally, there was a moderate negative correlation between inspection latency and the number of individuals caught within a given trap because traps with shorter inspection latencies captured more individuals (Pearson: *r* = −0.384, *p* = 0.044; Figure 7b). Similarly, there was also a negative correlation between latency to enter a trap and the number of individuals caught by a trap (Pearson: *r* = −0.429, *p* = 0.05; Figure 7c).

### 3.4 Inspection and entry rate

**FIGURE 8:**
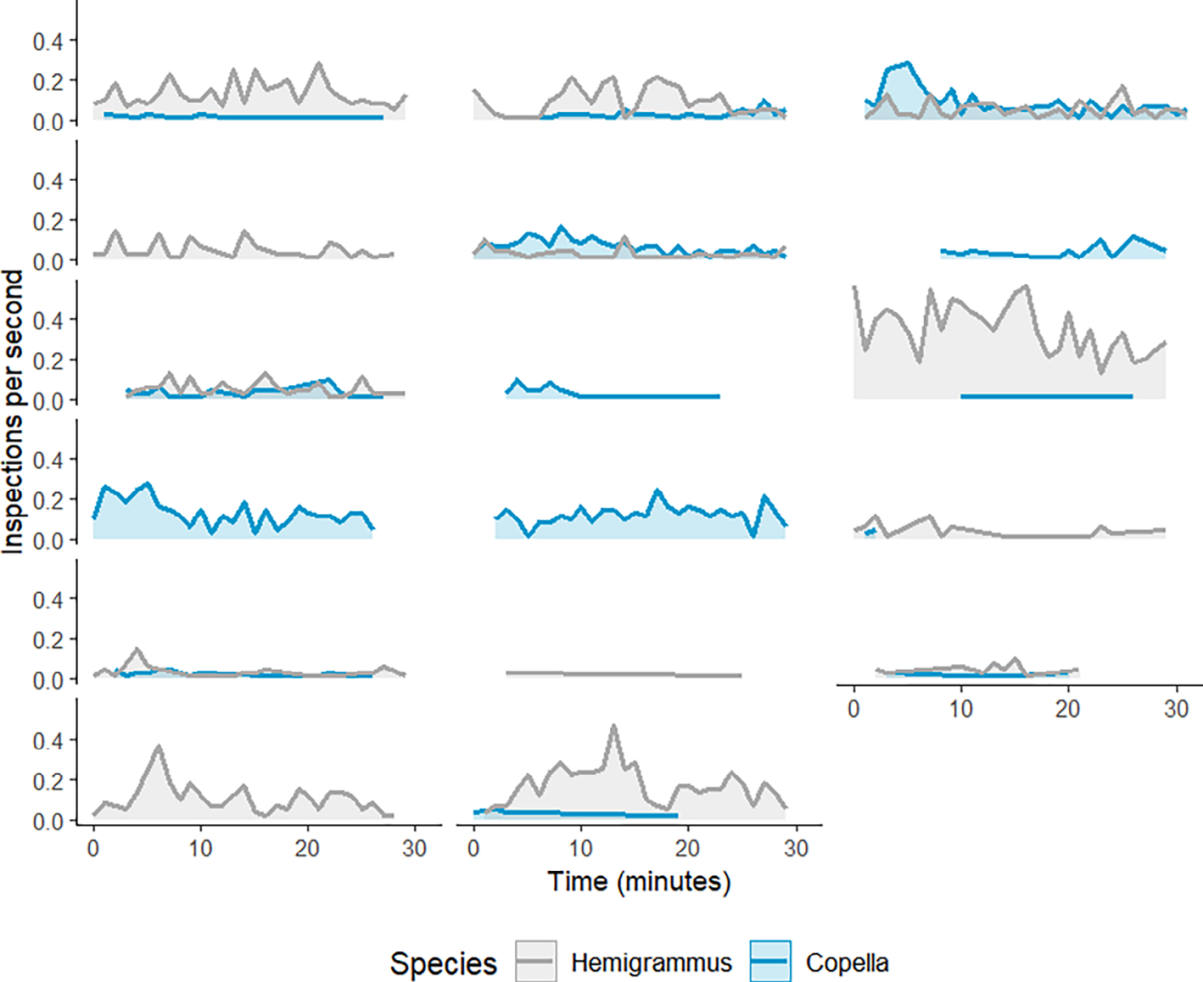
The rate of inspection of a trap over a 30‐min trial. Each panel represents an individual trial, with each line displaying the peaks and troughs in the rate of inspection over the entire trial. The *y* axis represents the inspections per seconds and the *x* axis represents the time within the 30‐min trial. The colour of the line corresponds to the species.

Inspection rate differed within and across trials (Figure 8). Inspections were observed in 17 of the 18 trials that were used for video analysis. Five trials had inspections for only one species while the remaining trials had inspections for both. Within trials, the rate of inspection over time varied. While some trials had a peak in activity at the start, other trials had a peak towards the end of the trapping period, and others had a continuous rate of activity. This pattern also differed across trials and the rate of inspection also differed between species across trials. However, the level of activity within a trial was usually not high for both species simultaneously, with one species tending to have a greater level of activity over the other.

**FIGURE 9:**
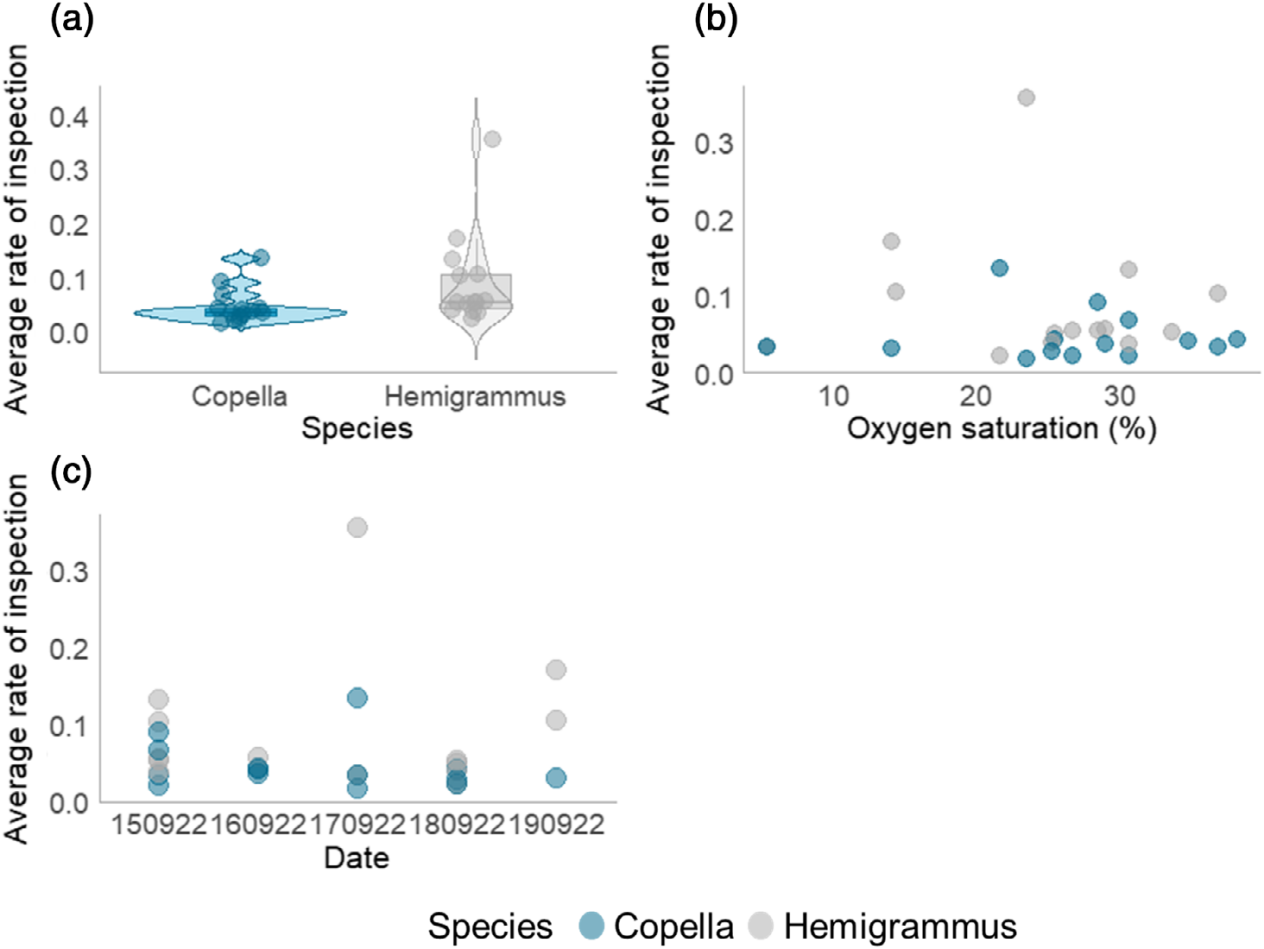
The impact of predictor variables on the average rate of inspection: (a) the difference in the rate of inspection between species; (b) the relationship between rate of inspection and oxygen saturation; (c) the average rate of inspection across trapping dates.

Inspection rate was affected by several factors. For example, there is strong evidence that both date of capture (*t* = 3.064, *p* = 0.006; Figure 9c) and species (*t* = 2.450, *p* = 0.023; Figure 9a) can explain variation in the rate of inspection, with higher rates of inspection for Hemigrammus (Table S5). There was also moderate evidence that dissolved oxygen influenced the inspection rate, with higher rates of inspection at higher dissolved oxygen concentrations (*t* = 2.505, *p* = 0.021; Figure 9b). Additionally, there was a positive correlation between the number of individuals caught within a trap and the rate of inspection (Pearson: *r* = 0.668, *p* < 0.001).

**FIGURE 10:**
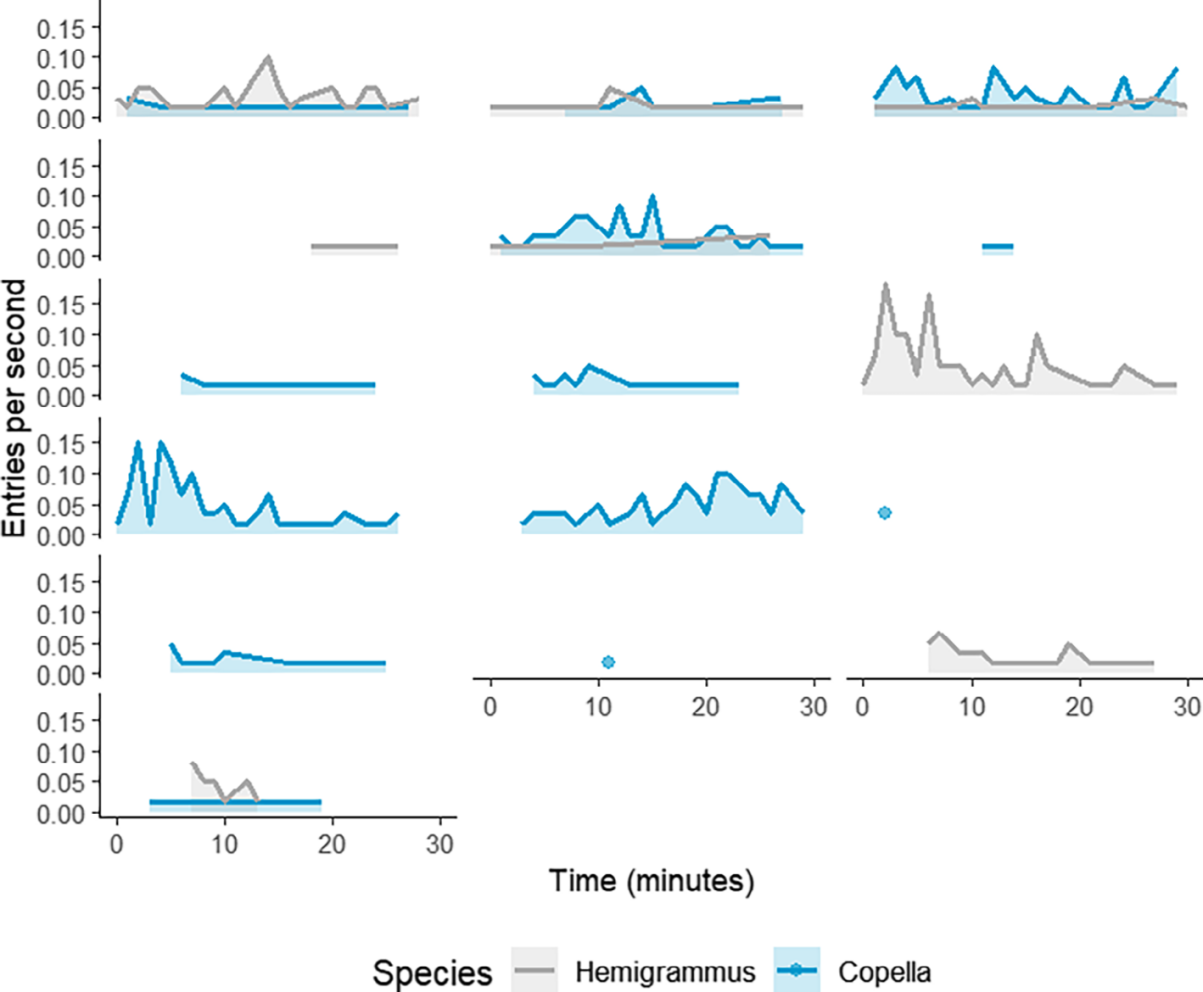
The rate of entry of a trap over a 30‐min trial. Each panel represents an individual trial, with each line displaying the peaks and troughs in the rate of entry over the 30‐min trial. Panels with a single data point indicate that only one entry occurred. The *y* axis represents the entries per second and the *x* axis represents the time within the 30‐min trial. The colour of the line corresponds to the species.

The entry rate also differed across and within trials. Entries were observed in 16 of the 18 and 68.8% of the trials had entries for only one species. Within trials, the entry rate also differed, with some trials having peaks of entries at the start of the trial and others having peaks of activity occurring throughout the duration of the trial (Figure 10). Furthermore, while variation in the entry rate could be explained by date of capture (*t* = 4.145, *p* < 0.001), there was little evidence that any of the other predictor variables had an impact on the entry rate (Table S5). However, there was a strong positive correlation between the rate of entry and the number of individuals caught by a trap (Pearson: *r* = 0.888, *p* < 0.001).

### 3.5 Sociability

**FIGURE 11:**
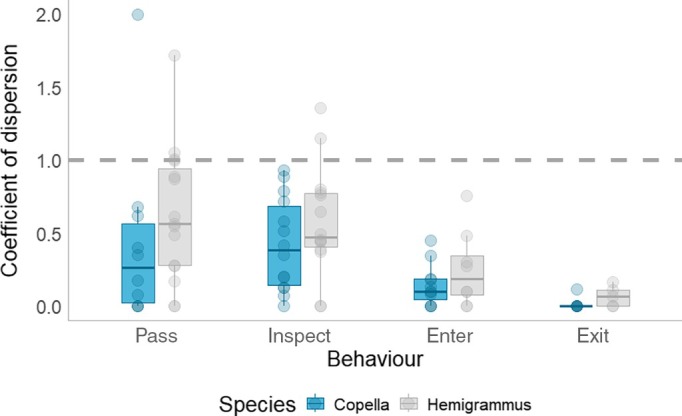
The coefficient of dispersion of four behaviours (passes, inspections, entries and exits) for each trial and for both species.

Clustering in the timing of behavioural events was found in only six trials (Figure 11). When synchronised behaviours did occur, it was only for passes and inspections. There was also evidence overall for a difference in the CD between behaviours, with CD declining between behaviours (from pass to entry). There was also evidence for differences in CD between species, with Hemigrammus having a higher CD for all behaviours (Figure 11 and Table S6).

**FIGURE 12:**
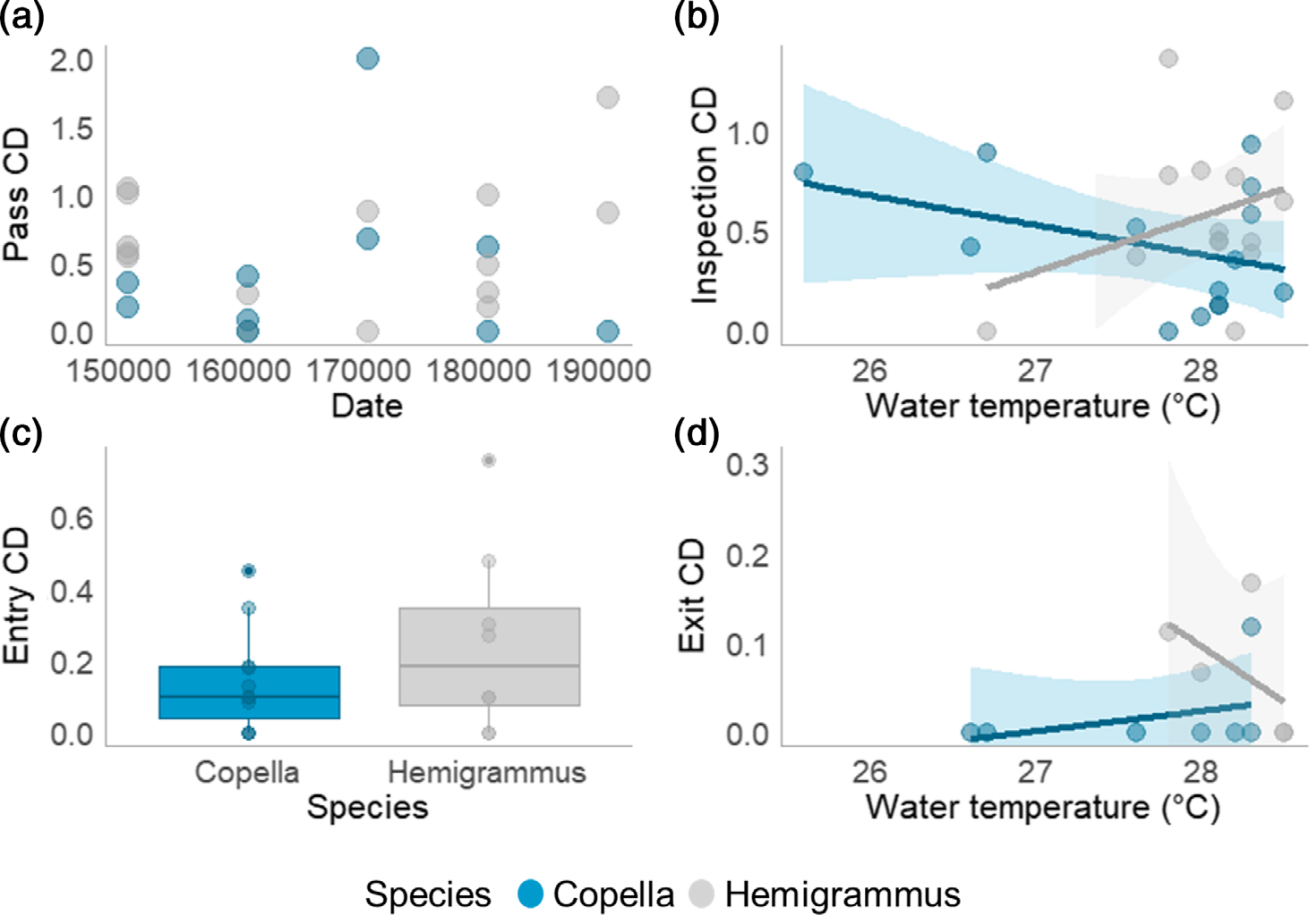
The impact of model predictors on the coefficient of dispersion (CD) of four behaviours (passes, inspections, entries and exits) for both species. Each data point represents the overall CD for passes within a trial: (a) the impact of date of capture on the CD of passes; (b) the impact of water temperature on the CD of inspections; (c) the impact of location on the CD of entries; and (d) the impact of water temperature on the CD of exits.

Several factors influenced the degree to which behaviours were temporally clustered. For passes, there was moderate evidence that date of capture influenced CD (*t* = 2.365, *p* = 0.034; Figure 12a). Additionally, there was moderate evidence that water temperature influenced the CD of inspections (*t* = 2.309, *p* = 0.032). However, this was dependent on species, with greater CDs at higher temperatures for Hemigrammus, but the opposite trend for Copella (Figure 12b). Similarly, the effect of water temperature on CD for exits varied between species (Table S6), with higher CDs at elevated temperatures for Copella rather than Hemigrammus (*t* = 4.657, *p* = 0.01; Figure 12d). Finally, there was no evidence that any of the predictor variables apart from species (Table S6) had any impact on entry CD (*t* = 2.215, *p* = 0.049; Figure 12c).

## 4 DISCUSSION

Understanding the relationship between an individual's traits and its susceptibility to capture is crucial for unravelling the mechanisms through which interactions with fishing gears can lead to changes in a targeted population (Hollins et al., 2018). The capture process involves many steps and identifying behavioural mechanisms underlying capture is essential to determine which traits are under selective pressure from fisheries (Ward & Webster, 2016). Furthermore, as physiological and behavioural traits related to capture are also closely linked with environmental factors (Hollins et al., 2018), it is important to understand how changes in environment can modulate behavioural responses to gears. Here, we demonstrate that observations of traps in the wild provide insights not only into the number of individuals caught, an endpoint often focussed on by previous studies (Hollins et al., 2019; Thambithurai et al., 2018; Thambithurai et al., 2022), but also into pre‐capture behaviours, such as passes and inspections. While this study did not directly measure phenotypic traits outside of the capture process, insights into behavioural decision‐making shed light on which behaviours influence capture and which do not. Additionally, we reveal how environmental factors, such as temperature and dissolved oxygen, influence how individuals approach and exit traps, and how the relationship between behaviour and environment can differ between species.

While a range of Amazonian species such as *Apistogramma* spp. and *Nannostomus* spp. were spotted around the vicinity of the traps (Table S2), Copella and Hemigrammus dominated trap interactions. Despite the presence of non‐target species, it is not believed that their presence impacted our focal species due to their low abundance and because they are typically found within the same habitat (Machado et al., 2020). Furthermore, only a small fraction of individuals that passed by or inspected traps ultimately entered them, a pattern consistent with findings from commercial food fisheries (Anders et al., 2017; Meintzer et al., 2017; Rose et al., 2005). The fact that only a small proportion of fish that passed by or inspected the trap actually entered suggests that there may be strong potential for selection for particular traits that influence trap entry, which differ between those that choose to enter the trap and the many that do not. Not only did a small proportion of fish enter the trap, but this was also not correlated with the number of inspections. For example, one trial saw 600 inspections compared to the usual mean of 80, yet no increase in the number of entries. The discrepancy between entries and inspections suggests that other factors, beyond the number of fish around a trap, ultimately influence the number of fish that enter and are captured.

Contrary to findings from other studies, such as Anders et al. (2017), which saw a difference in trap vulnerability between gadoid species in northern Norway, we did not find a difference in overall capture rates between the two species. However, within a trial, traps tended to catch one species disproportionately. Therefore, while traps have the capacity to attract multiple species, there may be differences between the focal species in environmental preference, interspecific variation in attraction to conspecific cues or local variation in abundance, which can contribute to a given species being captured. Furthermore, while predator presence may have influenced our focal species, we found no evidence of predatory fish in our videos or during field sampling.

Interestingly, environmental variables such as temperature and dissolved oxygen did not affect the number of fish captured or the frequency of pre‐capture behaviours like passes or inspections. This contrasts with studies such as Clark and Ioannou (2025), which found a greater number of captured sticklebacks in warmer water. However, date of capture significantly predicted the number of passes in our study, which may indirectly reflect changes in local environment or the composition of the population on a particular day. While the underlying mechanism remains inconclusive, it highlights how daily variation can have important repercussions for fisheries.

We found no difference between species in inspection or entry latency. The lack of a difference here could indicate that both species may have similarities in traits that determine the timing of an approach or entry. However, while there was no difference between species, water temperature played a key role as individuals inspected faster at higher temperatures. This finding aligns with studies that have linked temperature and boldness (Biro et al., 2009; Forsatkar et al., 2016) and may be a function of increased activity at higher temperatures (Bartolini et al., 2015; Tiddy et al., 2024), which can increase the likelihood of an individual to encounter a passive gear (Hollins et al., 2018; Thomsen et al., 2010).

Previous research on commercial traps reveals a trend in the rate of entries (reviewed by Thomsen et al., 2010), with entries increasing, then levelling off, before declining. However, our results indicate that in artisanal fisheries, the rate of inspection and entry fluctuates. A reason for this difference could be that compared to commercial food traps, the artisanal traps used in this study were left for shorter periods, although this is more typical of the trap durations used by ornamental fishers. Nevertheless, we did find that the rate of inspection increased at higher dissolved oxygen levels, suggesting that environmental factors, particularly for fisheries within the Amazon, is an important driver of behaviour.

Very few behaviours around traps were temporally clustered, with synchronisation observed only for passes and inspections. The lack of coordination in entries challenges the assumption that sociability increases vulnerability to capture (Hollins et al., 2018; Thambithurai et al., 2018). Both Hemigrammus and Copella are schooling species (Ashraf et al., 2016), but the observed individual‐level responses suggest that sociability may play a lesser role in final entry decisions than previously thought and that decisions to enter are made as individuals. This individual behaviour opens the door for selection to act on other components of behaviour at the individual level without social constraints operating.

Species‐specific responses to temperature were particularly striking in the context of coordination. In Hemigrammus, the CD of inspections increased with water temperature, indicating more coordinated behaviour, while the opposite was true for Copella. Conversely, Copella displayed greater coordination in exits (higher CD) at higher temperatures, while the opposite trend was found for Hemigrammus. These contrasting patterns may reflect species‐specific differences in how temperature influences activity levels, social behaviour or behavioural plasticity. For example, increased water temperature may enhance group‐level responsiveness in Hemigrammus, leading to more coordination when inspecting a trap, whereas Copella may exhibit more individual or variable responses. While the mechanisms underlying these contrasting patterns remains unclear, the variation in behaviour of the two species suggests that environmental changes may selectively affect trap vulnerability and behaviour in species‐specific ways.

Importantly, our findings represent a snapshot of the complex environmental dynamics within the Amazon. Our study was conducted in the dry season, when increases in water temperature and reductions in dissolved oxygen levels are part of expected seasonal variation (Junk & Soares, 2010). However, as seasonal extremes are becoming more pronounced due to anthropogenic pressures (Espinoza et al., 2024; Ottoni et al., 2023), it is important to understand how changes in environmental conditions can modulate relationships between species‐specific behavioural responses and their vulnerability to capture.

## 5 CONCLUSION

Our study aimed to understand how fish behave around artisanal traps in the wild, how environmental factors influence the capture process, and whether differences could be observed between two Amazonian species. We found that the behaviour of fish around traps in the wild is complex, with only a fraction of fish that pass by or inspect a trap actually entering. While we found no difference in the capture rate or latency to inspect or enter a trap between species, traps typically caught one species over the other and we observed species‐specific variation in the timing and coordination of behavioural events, which was further influenced by environmental factors. Indeed, while environmental factors had no impact on the number of individuals caught, the time taken for an individual to inspect a trap was shorter at higher temperatures and the rate of inspection increased at higher oxygen saturation levels. Importantly, the relationship between environmental factors and the coordination of behaviours varied between species. While warmer temperatures led to more coordinated inspections, but less coordinated exits for Hemigrammus, the opposite was true for Copella. Overall, our findings demonstrate that using a low‐cost method to observe behaviours in uncontrolled settings provides a valuable insight into how traps work, particularly in artisanal settings. Our findings also provide a more comprehensive understanding of the behaviours involved in the capture process, including pre‐capture behaviours, and how environmental factors can drive differences between species.

## AUTHOR CONTRIBUTIONS

M.P., S.K. and D.K. designed the study. M.P., S.K., D.K. and J.L.G.M. generated field data. M.P. carried out video and statistical analysis. M.P. drafted the initial manuscript. M.P., S.K., D.K., J.L., K.E. and A.L.V. contributed towards the final draft of the manuscript. Funding was provided by M.P., S.K., D.K. and A.L.V.

## FUNDING INFORMATION

This work was supported by funds from the Fisheries Society of the British Isles, Fundação de Amparo à Pesquisa do Estado do Amazonas and Conselho Nacional de Desenvolvimento Científico e Tecnológico supporting INCT ADAPTA (CNPQ process 465540/2014‐7 and FAPEAM process 062.01187/2017).

## Supporting information


**DATA S1** Supporting Information.

## Data Availability

Data supporting this study are openly available from Mendeley Data at https://doi.org/10.17632/7f9g68ryjb.1.
