# Supplementary material for: Species‐specific behaviour and environmental drivers of trap interactions in wild ornamental fishes
Source: J Fish Biol. 2025 Sep 7;107(6):2092–105. doi: 10.1111/jfb.70217 (PMC12861839; doi:10.1111/jfb.70217)
Supplement: Supplementary file 1 — DATA S1 Supporting Information. [file JFB-107-2092-s001.docx]

**SUPPLEMENTARY MATERIAL**

Table 1: Surveying Schedule

**Table 1** Fieldwork schedule with number of days fished at each site

| Date | Site | Number of days fishing in site |
| --- | --- | --- |
| 15/09/2022 | 1 | 1 |
| 16/09/2022 | 1 | 2 |
| 17/09/2022 | 2 | 1 |
| 18/09/2022 | 2 | 2 |
| 19/09/2022 | 3 | 1 |

Figures 1-2: Environmental Data


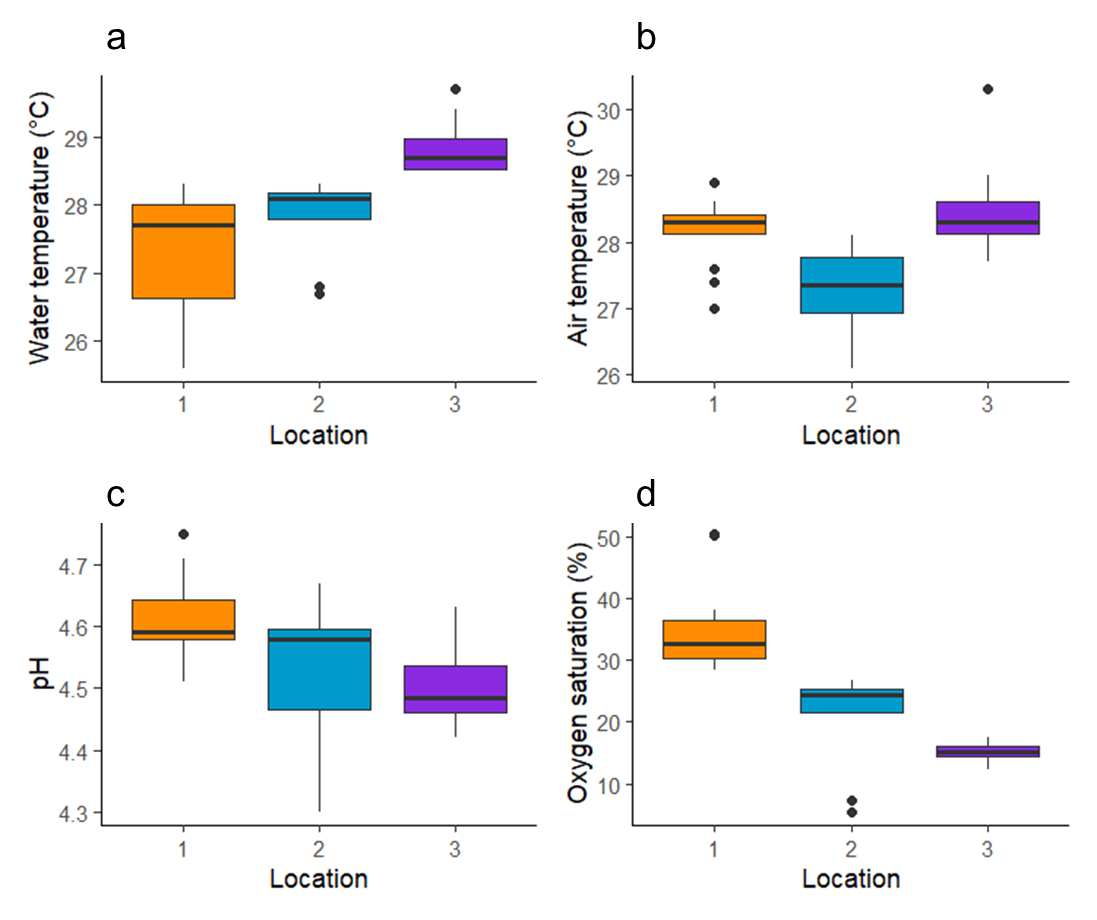


**Figure 1** Boxplots for environmental data across the three field sites: a) water temperature; b) air temperature; c) pH; and d) oxygen saturation.


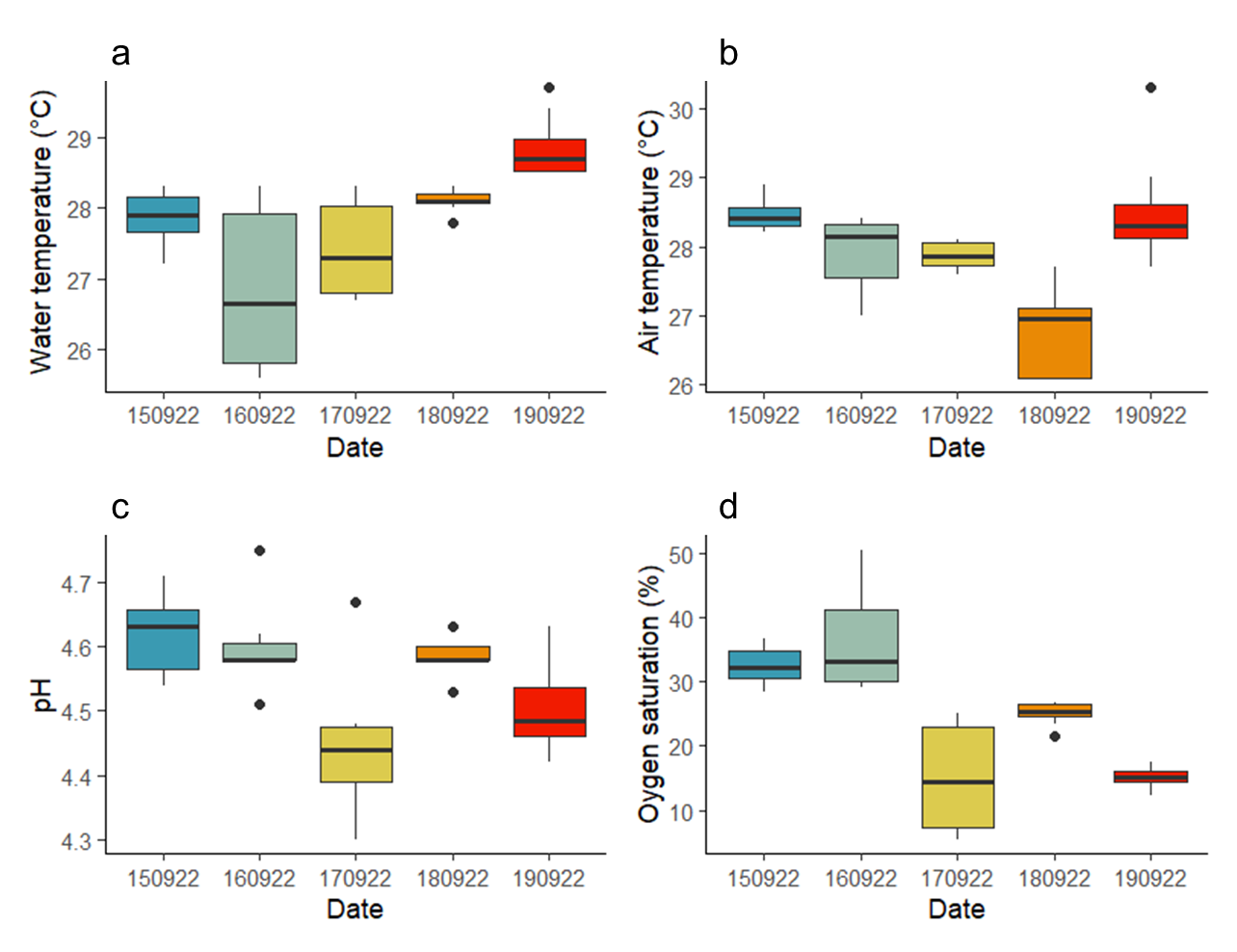


**Figure 2** Boxplots for environmental data on different survey dates: a) water temperature; b) air temperature; c) pH; and d) oxygen saturation.

Table 2: Taxonomic List

**Supplementary Table 2** Taxonomic list of all identified genus and species visible within the field of view for each video trial

| Date | Site | Trial | Genus/ Species |
| --- | --- | --- | --- |
| 15/09/2022 | 1 | 1 | *Copella nattereri, Hemigrammus sp., Nannostomus eques* |
| 15/09/2022 | 1 | 3 | *Apistogramma sp., Copella nattereri, Hemigrammus sp.* |
| 15/09/2022 | 1 | 4 | *Copella nattereri, Hemigrammus sp.* |
| 15/09/2022 | 1 | 5 | *Copella nattereri, Hemigrammus sp.* |
| 15/09/2022 | 1 | 6 | *Apistogramma sp., Copella nattereri, Hemigrammus sp., Bryconops sp. (larval)* |
| 16/09/2022 | 1 | 1 | *Apistogramma sp., Mesonauta insignis* |
| 16/09/2022 | 1 | 2 | *Copella nattereri, Bryconops sp. (larval)* |
| 16/09/2022 | 1 | 3 | *Copella nattereri, Hemigrammus sp., Nannostomus eques.* |
| 16/09/2022 | 1 | 5 | *Apistogramma sp., Copella nattereri, Bryconops sp. (larval), Mesonauta insignis* |
| 17/09/2022 | 2 | 1 | *Bryconops sp., Copella nattereri, Hemigrammus sp., Hemigrammus sp. 2., Nannostomus eques* |
| 17/09/2022 | 2 | 3 | *Copella nattereri* |
| 17/09/2022 | 2 | 5 | *Copella nattereri, Hemigrammus sp.* |
| 18/09/2022 | 2 | 1 | *Copella nattereri, Hemigrammus sp.* |
| 18/09/2022 | 2 | 3 | *Copella nattereri, Hemigrammus sp.* |
| 18/09/2022 | 2 | 5 | *Bryconops sp., Hemigrammus sp., Nannostomus eques* |
| 18/09/2022 | 2 | 7 | *Bryconops sp., Copella nattereri, Hemigrammus sp.* |
| 19/09/2022 | 3 | 1 | *Apistogramma sp., Hemigrammus sp., Nannostomus eques* |
| 19/09/2022 | 3 | 5 | *Hemigrammus sp., Moenkhausia cotinho, Nannostomus eques* |

Figure 3: Location of Entry


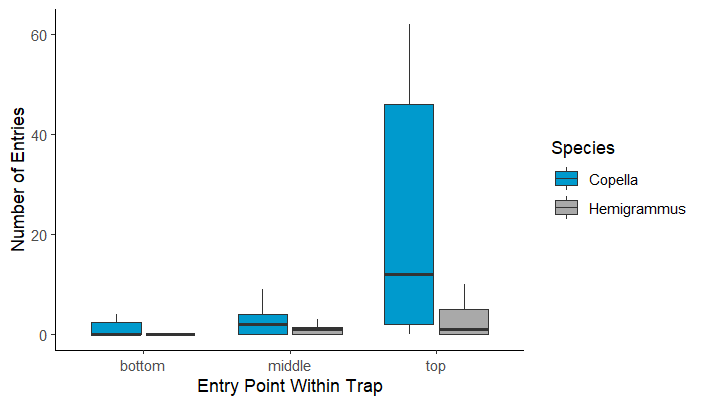


**Figure 3** Number of entries within different locations of the trap for both species using data from high visibility videos


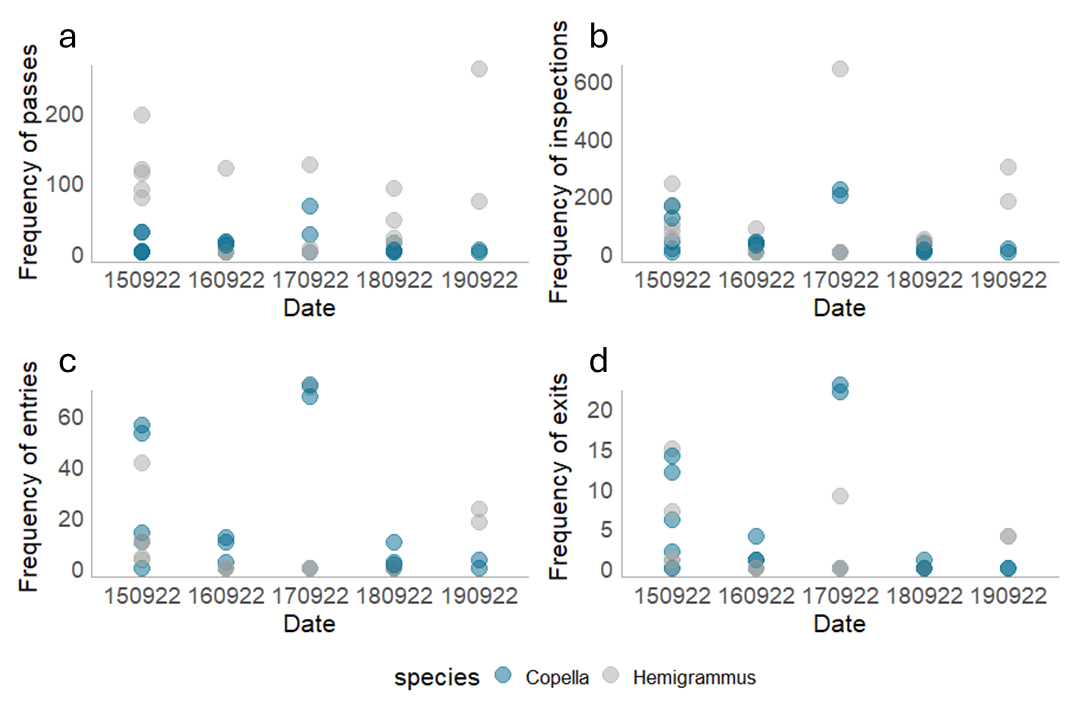


**Figure 4** The relationship between date of capture and the frequency of: a) passes; b) inspections; c) entries; and d) exits.

Table 3: Model estimates for number of fish caught and frequency of passes, inspections, entries, and exits

**Table 3** Summary of factors contributing to the frequency of observed behaviours (passes, inspections, entries and exits) and the number of individuals caught (entries-exits). Each model shown is the final model determined via stepwise model selection (as described in methods). The multiple R-squared (r^2^_m_) and adjusted R-squared (r^2^_a_) are also shown for each model. The threshold level of significance for p values is given at 0.05 and significant values are displayed in bold.

|  | Estimate | Std. Error | t value | Pr (>\|t\|) | r^2^_m_ | r^2^_a_ |
| --- | --- | --- | --- | --- | --- | --- |
| Pass frequency |  |  |  |  | 0.4561 | 0.3436 |
| Intercept  date160922  date170922  date180922  date190922  trial  Species Hemigrammus | 57.957  -51.960  -33.374  -40.073  14.043  -6.509  63.556 | 24.901  24.151  26.026  23.687  29.747  4.572  16.633 | 2.328  -2.151  -1.282  -1.692  0.472  -1.424  3.821 | **0.027**  **0.040**  0.210  0.101  0.640  0.165  **< 0.001** |  |  |
| Inspection frequency |  |  |  |  | 0.2731 | 0.1519 |
| Intercept  date160922  date170922  date180922  date190922  Species Hemigrammus | 67.07  -73.03  81.10 -75.78  24.85  57.67 | 41.28  54.78  59.64  54.78  68.33  38.50 | 1.624  -1.333  1.360  -1.383  0.364  1.498 | 0.115  0.193  0.184  0.177  0.719  0.145 |  |  |
| Entry frequency |  |  |  |  | 0.303 | 0.2131 |
| Intercept  date160922  date170922  date180922  date190922 | 20.200  -17.200  14.800  -18.575  -9.200 | 6.245  9.367  10.198  9.367  11.683 | 3.235  -1.836  1.451  -1.983  -0.787 | **0.003**  0.076  0.157  0.056  0.437 |  |  |
| Exit frequency |  |  |  |  | 0.2908 | 0.144 |
| Intercept  date160922  date170922  date180922  date190922  Species Hemigrammus  Oxygen saturation (%) | -59.845  -76.991  141.251  -46.922  95.246  57.667  3.966 | 154.766  55.234  92.651  64.639  107.482  38.676  4.660 | -0.387  -1.394  1.525  -0.726  0.886  1.491  0.851 | 0.702  0.174  0.138  0.474  0.383  0.147  0.402 |  |  |
| Caught |  |  |  |  | 0.2868 | 0.1948 |
| Intercept  date160922  date170922  date180922  date190922 | 14.400  -12.275  11.600  -12.900  -5.400 | 4.745  7.117  7.748  7.117  8.877 | 3.035  -1.725  1.497  -1.812  -0.608 | **0.005**  0.095  0.145  0.080  0.548 |  |  |

Table 4: Model estimates for latency

**Table 4** Summary of factors contributing to the inspection latency and entry latency. Each model shown is the final model determined via stepwise model selection (as described in methods). The multiple R-squared (r^2^_m_) and adjusted R-squared (r^2^_a_) are also shown for each model. The threshold level of significance for p values is given at 0.05 and significant values are displayed in bold.

|  | Estimate | Std. Error | t value | Pr (>\|t\|) | r^2^_m_ | | r^2^_a_ |
| --- | --- | --- | --- | --- | --- | --- | --- |
| Inspection latency |  |  |  |  | | 0.2532 | 0.2245 |
| Intercept  Water temperature | 3385.10  -116.14 | 1090.48  39.11 | 3.104  -2.969 | **0.005**  **0.006** | |  |  |
| Entry latency |  |  |  |  | |  |  |
| Intercept | 276.83 | 62.48 | 4.431 | **<0.001** | |  |  |

Table 5: Model estimates for rate of inspection and entry

**Table 5** Summary of factors contributing to the average rate of inspection and entry. Each model shown is the final model determined via stepwise model selection (as described in methods). The multiple R-squared (r^2^_m_) and adjusted R-squared (r^2^_a_) are also shown for each model. The threshold level of significance for p values is given at 0.05 and significant values are displayed in bold.

|  | Estimate | Std. Error | t value | Pr (>\|t\|) | r^2^_m_ | r^2^_a_ |
| --- | --- | --- | --- | --- | --- | --- |
| Average rate of inspection |  |  |  |  | 0.4644 | 0.3114 |
| Intercept  date160922  date170922  date180922  date190922  Species Hemigrammus  Oxygen saturation (%) | -0.163  -0.010  0.157  0.011  0.140  0.055  0.006 | 0.084  0.035  0.051  0.033  0.058  0.022  0.002 | -1.944  -0.301  3.064  0.348  2.423  2.450  2.505 | 0.065  0.766  **0.006**  0.731  **0.025**  **0.023**  **0.021** |  |  |
| Average rate of entry |  |  |  |  | 0.5859 | 0.4824 |
| Intercept  date160922  date170922  date180922  date190922 | 0.026  -0.006  0.023  -0.001  0.004 | 0.003  0.006  0.006  0.006  0.006 | 9.166  -1.041  4.145  -0.240  0.655 | **<0.001**  0.313  **<0.001**  0.814  0.522 |  |  |

Table 6: Model estimates for sociability

**Table 6** Summary of factors contributing to the coefficient of dispersion. Each model shown is the final model determined via stepwise model selection (as described in methods). The multiple R-squared (r^2^_m_) and adjusted R-squared (r^2^_a_) are also shown for each model. The threshold level of significance for p values is given at 0.05 and significant values are displayed in bold.

|  | Estimate | Std. Error | t value | Pr (>\|t\|) | r^2^_m_ | r^2^_a_ |
| --- | --- | --- | --- | --- | --- | --- |
| CD pass |  |  |  |  | 0.6755 | 0.4009 |
| Intercept  date160922  date170922  date180922  date190922  species Hemigrammus  water temperature  date160922 * species Hemigrammus  date170922 * species Hemigrammus  date180922 * species Hemigrammus  species Hemigrammus * water temperature | 6.538  -0.352  0.977  0.081  -0.137  -11.620  -0.225  -0.051  -1.138  -0.383  0.564  0.433 | 4.950  0.419  0.413  0.406  0.506  7.250  0.177  0.573  0.552  0.489  0.617  0.259 | 1.321  -0.840  2.365  0.200  -0.270  -1.603  -1.270  -0.088  -2.063  -0.783  0.914  1.673 | 0.209  0.416  **0.034**  0.845  0.791  0.133  0.226  0.931  0.060  0.448  0.377  0.118 |  |  |
| CD inspect |  |  |  |  | 0.4092 | 0.2024 |
| Intercept  date160922  date170922  date180922  date190922  species Hemigrammus  water temperature  species Hemigrammus * water temperature | 3.190  -0.005  0.264  -0.297  -0.030  -16.106  -0.100  0.583 | 3.416  0.219  0.192  0.160  0.226  7.059  0.122  0.252 | 0.934  -0.022  1.377  -1.861  -0.133  -2.282  -0.815  2.309 | 0.361  0.983  0.184  0.078  0.896  **0.034**  0.425  **0.032** |  |  |
| CD enter |  |  |  |  | 0.473 | 0.579 |
| Intercept  location tabazinho  location yacaremiri  date160922  date170922  species Hemigrammus  location tabazinho * species Hemigrammus  location yacaremiri * species Hemigrammus | 0.100  0.024  -0.100  -0.062  0.298  0.357  -0.346  -0.070 | 0.132  0.147  0.186  0.101  0.161  0.161  0.184  0.228 | 0.759  0.163  -0.537  -0.625  1.849  2.215  -1.879  -0.307 | 0.464  0.873  0.602  0.545  0.092  **0.049**  0.087  0.765 |  |  |
| CD exit |  |  |  |  | 0.978 | 0.940 |
| Intercept  date160922  date170922  date190922  species Hemigrammus  water temperature  oxygen saturation  species Hemigrammus * water temperature | -1.109  0.044  0.129  -0.195  -8.482  0.036  0.003  0.306 | 0.530  0.039  0.028  0.032  1.839  0.021  0.002  0.066 | -2.093  1.127  4.666  -6.127  -4.611  1.766  1.438  4.657 | 0.105  0.323  **0.010**  **0.004**  **0.010**  0.152  0.224  **0.010** |  |  |
